# Rate Optimization of Intelligent Reflecting Surface-Assisted Coal Mine Wireless Communication Systems

**DOI:** 10.3390/e26100880

**Published:** 2024-10-20

**Authors:** Yang Liu, Zhao Yang, Bin Wang, Yanhong Xu

**Affiliations:** School of Communication and Information Engineering, Xi’an University of Science and Technology, Xi’an 710054, China; yangz@stu.xust.edu.cn (Z.Y.); wangbin@mail.xidian.edu.cn (B.W.); yhxu@xust.edu.cn (Y.X.)

**Keywords:** intelligent reflecting surface, achievable rates, rate optimization, coal mine wireless communication system

## Abstract

This paper proposes a three-step joint rate optimization method for intelligent reflecting surface (IRS)-assisted coal mine wireless communication systems. Different from terrestrial IRS-assisted communication scenarios, in coal mines, IRSs can be installed flexibly on the tops of rectangular tunnels to address the issues of signals being blocked and interfered with by mining equipment. Therefore, it is necessary to optimize the IRS deployment position, the transmit power and IRS phase shifts to achieve the maximum effective achievable rate at user stations equipped with the proposed system. However, due to the complex channel models of coal mines, the optimization problem of IRS deployment position is non-convex. To solve this problem, two auxiliary variables along with logarithmic operations and Taylor approximation are introduced. On this basis, a three-step joint rate optimization involving the transmit power, IRS phase shifts and IRS deployment position is proposed to maximize the effective achievable rates at the user station. The simulation results show that compared with other rate optimization schemes, the effective achievable rates at the user station using the proposed joint rate optimization scheme can be improved by approximately 12.32% to 54.17% for different parameter configurations. It is also pointed out that the deployment position of the IRS can converge to the same optimal position independent of the initial deployment position. Moreover, we investigate the effects of the roughness of the tunnel walls in a coal mine on the effective achievable rates at the user station, and the simulation results indicate that the proposed three-step joint rate optimization scheme performs better in the coal mine scenario regardless of the roughness.

## 1. Introduction

Coal mines, as a vertical industrial application of 5th/6th-Generation (5G/6G) wireless communication networks, face critical challenges due to poor wireless propagation environments, such as uneven and rough tunnel walls, coal dust and high humidity, obstructions from mining equipment (ME) and so on [1]. These adverse factors result in complex and special wireless transmission characteristics in coal mines, including severe transmission loss, signal attenuation and distortion, and multipath interference. In particular, ME in coal mines will block direct communication links and degrade efficient signal transmission, which will create potential safety risks [2,3,4]. Traditional relay solutions have the issues of higher costs and power consumption, more complicated maintenance and lower efficiency. Therefore, there is an urgent need for new wireless communication technology with the attributes of low power consumption, low costs and high reliability to overcome these challenges and improve the quality of coal mine wireless communication systems.

As a candidate technology of 6G, intelligent reflecting surfaces (IRSs) have emerged due to their intelligent manipulation of the wireless propagation environment, flexible reconfiguration, low power consumption and cost and high spectral and energy efficiency, providing an innovative and effective means to realize excellent communication by directly reshaping wireless propagation channels in favor of signal transmission [5,6,7,8,9]. Since IRSs are economical and easy to deploy in the tunnels of coal mines and can address signals that are blocked and interfered with by obstructions, they have promise for applications in coal mines to enhance communication metrics [10]. Among them, the effective achievable rate, as a crucial metric of coal mine communication systems, plays a vital role in ensuring safety in a coal mine.

Much work has focused on the rate optimization of various IRS-assisted communication scenarios. In [11], the joint optimization of active transmit and passive reflective beamforming is investigated. High-quality suboptimal solutions are obtained by alternating optimization, semi-definite relaxation (SDR) and Gaussian randomization to maximize the target rate. To reduce the computational complexity of Gaussian randomized SDR, a simple neural network is proposed to replace SDR at the cost of a reduced secrecy rate [12]. Furthermore, the block coordinate ascent method (BCAM) is employed to find stationary solutions to the formulated non-convex optimization problem, which can reduce computational complexity to linear time with the number of IRS elements without compromising the secrecy rate too much [13]. These works only performed the joint optimization of transmit power and IRS phase shifts, which are insufficient, as IRS deployments need to be involved in the optimization problem. In [14], it was proven that if the distance between the access point (AP) and user equipment (UE) is fixed, the IRS should be deployed near the AP or UE to achieve the maximum transmission rate in a single UE system. For an unfixed distance between the AP and UE, the strategy of IRS deployment positions in IRS-assisted wireless communication systems is theoretically analyzed and experimentally validated in [15], which shows that the optimal IRS deployment is related to the relationship between the distance from the AP to the UE and the distance from the IRS to the AP-UE line. Different from terrestrial communication scenarios, in underground coal mines, IRSs can usually be installed on the tops of tunnels to reduce the absorption and scattering of signals by various rock materials and address the issues of signals being blocked and interfered with by ME. Therefore, it is necessary to optimize the IRS deployment position to improve the effective achievable rate for IRS-assisted coal mine wireless communication systems.

In this paper, we propose a joint rate optimization involving transmit power, IRS phase shifts, and the IRS deployment position for an IRS-assisted coal mine wireless communication system with interference from ME to maximize the effective achievable rate at the user station. Specifically, we first develop an IRS-assisted coal mine communication channel model by considering the roughness of tunnel walls and scatters in the space based on a geometry-based stochastic model (GBSM), a 3rd-generation partnership project (3GPP) standardized modeling framework [16], to characterize a rectangular coal mine tunnel communication channel. Based on this, a three-step joint rate optimization scheme is proposed. Due to the confined tunnel construction in a coal mine, the IRS deployment position optimization becomes complex and non-convex. To address this issue, two auxiliary variables along with logarithmic operations and Taylor approximation are introduced into the optimization problem. In the simulations, we first validate the convergence of the proposed three-step joint rate optimization scheme and the analysis results show that the IRS deployment position can converge to the same optimal value independent of the initial IRS deployment position. Next, we also compare the effective achievable rates at the user station under the proposed three-step joint rate optimization scheme and other rate optimization schemes. The comparison results show that the effective achievable rates can be improved by approximately 12.32% and 54.17% by varying the total transmit power under different parameter configurations. Finally, we investigate the effects of the roughness of the tunnel walls of a coal mine on the effective achievable rates at the user station, and the simulation results indicate that the proposed three-step joint rate optimization scheme performs better in a coal mine scenario regardless of the roughness.

This paper is organized as follows. In Section 2, we establish an IRS-assisted wireless communication system model with the channel model based on a three-dimensional (3D) GBSM in a coal mine and formulate the joint rate optimization problem. In Section 3, we propose an optimization algorithm for the IRS deployment position and a three-step joint optimization scheme to maximize the effective achievable rate at the user station. In Section 4, we simulate and compare the results under the proposed rate optimization scheme and other schemes. In Section 5, we conclude our work.

## 2. System Model and Problem Formulation

### 2.1. Channel Modeling and System Model

**Figure 1:**
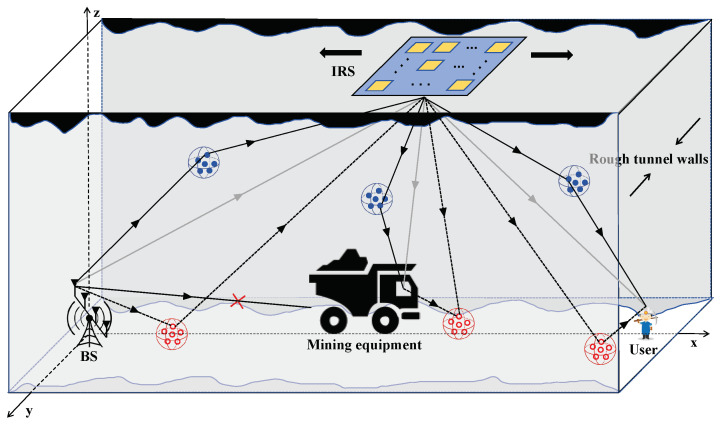
Illustration of 3D IRS-assisted coal mine wireless communication system model.

In this paper, we consider a rectangular coal mine tunnel and establish a coal mine channel model based on a 3D multiple input–multiple output (MIMO) GBSM under the 3GPP standard framework [17]. A three-dimensional description of an IRS-assisted coal mine wireless communication system is illustrated in Figure 1, which consists of a base station (BS) equipped with *K* antennas, an IRS with *N* low-cost passive reflecting elements, a single-antenna User and one single-antenna ME. In Figure 1, the gray lines represent the direct links between antenna pairs, and the red blank and blue solid circles represent the collections of multipath components generated by the reflection and refraction of the signal due to the roughness of the tunnel walls and scatters in the space. In this system model, the IRS is installed on the tops of tunnels and can be flexibly deployed along the horizontal axis, which forms a plane together with the BS, User and ME. We established a three-dimensional Cartesian coordinate system with the BS as the origin, denoting the coordinates of the BS, IRS, ME and User as b=[0,0,0], $I=[I_{x},I_{y},I_{z}]$, $m=[m_{x},m_{y},m_{z}]$ and $u=[u_{x},u_{y},u_{z}]$, respectively. We assume that $I_{y}$ = $m_{y}$ = $u_{y}$ = 0. Moreover, the direct link between the BS and the User is blocked by the mining equipment, which also interferes with the reflection link.

Next, we give a representation of the IRS-cascaded channel with ideal phase modulation combing using IRS technology. Here, it is assumed that the IRS phase modulation is ideal and independent of the angles of arrival in the BS-IRS and IRS–User channels. The IRS-cascaded channel between the BS and User can be represented as the products of the channel matrix of the BS-IRS link, the IRS phase shift matrix and the channel matrix of the IRS–User link, where both channel matrices can be modeled as the 3D MIMO GBSM for the coal mine [18].

Denote the channel matrix between the BS and IRS as $H^{BI}\left(f\right)\in C^{K\times N}$ and the entry of the *k*th row and *n*th column as $h_{kn}^{BI}\left(f\right)$ corresponding to the channel impulse response between the *k*th antenna of the BS and the *n*th reflecting element of the IRS. Here, C represents the complex-valued field. As shown in [18], the channel impulse response can be defined as the weighted sum of the Line-of-Sight (LoS) component and the Non-Line-of-Sight (NLoS) component. Therefore,

(1) $$h_{kn}^{BI}\left(f\right)=\sqrt{\frac{\kappa }{\kappa +1}}h_{kn}^{BI,L}\left(f\right)+\sqrt{\frac{1}{\kappa +1}}h_{kn}^{BI,NL}\left(f\right),$$

where κ is the Rician factor and *f* is the working frequency constrained by the carrier frequency $f_{c}$ and the bandwidth *B* within a range $\left[f_{c}-B/2,f_{c}+B/2\right]$, k=1,2,⋯,K and n=1,2,⋯,N.

The LoS component $h_{kn}^{BI,L}\left(f\right)$ is given by

(2) $$h_{kn}^{BI,L}\left(f\right)=\sqrt{\frac{C_{0}{\left(D_{kn}^{BI,L}\right)}^{-2}}{\sigma }\cdot }e^{j2\pi \tau_{kn}^{BI,L}\left(f_{c}-f\right)},$$

where $C_{0}$ represents the path loss for a propagation distance of 1 meter, $D_{kn}^{BI,L}$ is the distance of the LoS path between the *k*th antenna of BS and the *n*th reflecting element of IRS, σ is the noise standard deviation, $\tau_{kn}^{BI,L}$ is the propagation delay of the LoS component computed by $\tau_{kn}^{BI,L}=D_{kn}^{BI,L}/c$, *c* is the speed of light, and $f_{c}$ is the carrier frequency.

**Figure 2:**
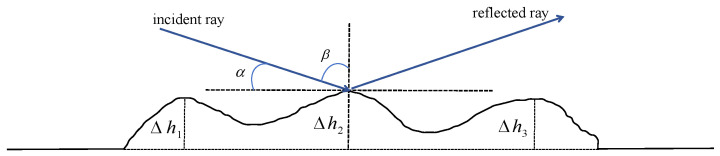
Effects of the roughness on the reflection phase.

The NLoS component $h_{kn}^{BI,NL}\left(f\right)$ is given by

(3) $$h_{kn}^{BI,NL}\left(f\right)=\sum_{m=1}^{M}\sqrt{\frac{C_{0}{\left(D_{kn,m}^{BI,NL}\right)}^{-2}}{\sigma }}\cdot \sqrt{P_{kn,m}^{BI,NL}}\cdot e^{j2\pi \tau_{kn,m}^{BI,NL}\left(f_{c}-f\right)}\cdot e^{-j\Delta \phi_{m}}\cdot \rho_{s}^{r_{m}},$$

where *M* denotes the total number of NLoS paths, $D_{kn,m}^{BI,NL}$ and $P_{kn,m}^{BI,NL}$ indicate the propagation distance and the normalized power of the *m*th NLoS path between the *k*th antenna of the BS and the *n*th reflecting element of the IRS, respectively. $\tau_{kn,m}^{BI,NL}$ is calculated as $D_{kn,m}^{BI,NL}/c$. $\Delta \phi_{m}$ represents the phase difference caused by the rough tunnel walls of the *m*th NLoS path and $\rho_{s}$ is the roughness attenuation factor. The reflection phase can be changed by the roughness of the coal mine tunnel wall surface as shown in Figure 2.

As shown in [19], the phase difference Δϕ is given by

(4) Δϕ=2rΔhsinα,

where Δh is the height variation of the rough surface which follows a Gaussian distribution, i.e., $\Delta h∼N(0,\delta_{h}^{2})$. A larger $\delta_{h}$ indicates a rougher surface. $r=2\pi f_{c}/c$ represents the number of waves and α is the angle between the incident ray and the horizontal plane.

The roughness attenuation factor can be expressed as

(5) $$\rho_{s}=\exp\left\{-8{\left(\frac{\pi \delta_{h}\cos\beta }{\lambda }\right)}^{2}\right\},$$

where β represents the angle between the incident ray and the vertical plane.

Similarly, the channel matrix between the IRS and User is denoted as $H^{IU}\left(f\right)\in C^{N\times 1}$ and the channel matrix between the IRS and ME as $H^{IM}\left(f\right)\in C^{N\times 1}$. The corresponding channel impulse responses $h_{n}^{IU}\left(f\right)$ between the *n*th reflecting element to the User and $h_{n}^{IM}\left(f\right)$ between the *n*th reflecting element to the ME can be given in analogy with Equation (1).

(6) $$h_{n}^{IU}\left(f\right)=\sqrt{\frac{\kappa }{\kappa +1}}h_{n}^{IU,L}\left(f\right)+\sqrt{\frac{1}{\kappa +1}}h_{n}^{IU,NL}\left(f\right),$$

where

(7) $$h_{n}^{IU,L}\left(f\right)=\sqrt{\frac{C_{0}{\left(D_{n}^{IU,L}\right)}^{-2}}{\sigma }\cdot }e^{j2\pi \tau_{n}^{IU,L}\left(f_{c}-f\right)}.$$

(8) $$h_{n}^{IU,NL}\left(f\right)=\sum_{m=1}^{M}\sqrt{\frac{C_{0}{\left(D_{n,m}^{IU,NL}\right)}^{-2}}{\sigma }}\cdot \sqrt{P_{n,m}^{IU,NL}}\cdot e^{j2\pi \tau_{n,m}^{IU,NL}\left(f_{c}-f\right)}\cdot e^{-j\Delta \phi_{m}}\cdot \rho_{s}^{r_{m}}.$$

(9) $$h_{n}^{IM}\left(f\right)=\sqrt{\frac{\kappa }{\kappa +1}}h_{n}^{IM,L}\left(f\right)+\sqrt{\frac{1}{\kappa +1}}h_{n}^{IM,NL}\left(f\right),$$

where

(10) $$h_{n}^{IM,L}\left(f\right)=\sqrt{\frac{C_{0}{\left(D_{n}^{IM,L}\right)}^{-2}}{\sigma }\cdot }e^{j2\pi \tau_{n}^{IM,L}\left(f_{c}-f\right)}.$$

(11) $$h_{n}^{IM,NL}\left(f\right)=\sum_{m=1}^{M}\sqrt{\frac{C_{0}{\left(D_{n,m}^{IM,NL}\right)}^{-2}}{\sigma }}\cdot \sqrt{P_{n,m}^{IM,NL}}\cdot e^{j2\pi \tau_{n,m}^{IM,NL}\left(f_{c}-f\right)}\cdot e^{-j\Delta \phi_{m}}\cdot \rho_{s}^{r_{m}}.$$

### 2.2. Problem Formulation

For simplicity, we consider that the distances for the different antenna pairs of the LoS path are almost the same, i.e., $D_{kn}^{BI,L}=D^{BI,L}$ for k=1,⋯,K and n=1,⋯,N in Equation (2). The same operations are made for other links, i.e., $D_{n}^{IU,L}=D^{IU,L}$ in Equation (7) and $D_{n}^{IM,L}=D^{IM,L}$ in Equation (10). Similarly, the distances for different NLoS paths are also almost equal and are linear to the distance of the LoS path, i.e., $D_{kn,m}^{BI,NL}=\epsilon D^{BI,L}$, $D_{n,m}^{IU,NL}=\epsilon D^{IU,L}$ and $D_{n,m}^{IM,NL}=\epsilon D^{IM,L}$ for m=1,2,⋯,M. For fixed coal mine tunnels, ϵ is generally set as (1,1.7]. Therefore, based on Equations (1)–(3), the channel matrix $H^{BI}\left(f\right)$ between BS and IRS can be expressed as

(12) $$H^{BI}\left(f\right)=\sqrt{\frac{C_{0}{\left(D^{BI,L}\right)}^{-2}}{\sigma }}\cdot \left(\sqrt{\frac{\kappa }{\kappa +1}}{\tilde{H}}^{BI,L}+\frac{1}{\epsilon }\sqrt{\frac{1}{\kappa +1}}{\tilde{H}}^{BI,NL}\right),$$

where ${\tilde{H}}^{BI,L}$ and ${\tilde{H}}^{BI,NL}$ represent the channel matrices constituted by the channel impulse responses of the LoS and NLoS components between the BS and IRS, excluding the constant coefficients. Here, $D^{BI,L}$ can be computed by

(13) $$D^{BI,L}=\sqrt{{∥b-I∥}^{2}}.$$

Similarly, the channel matrix $H^{IU}\left(f\right)$ between the IRS and User can be expressed as

(14) $$H^{IU}\left(f\right)=\sqrt{\frac{C_{0}{\left(D^{IU,L}\right)}^{-2}}{\sigma }}\cdot \left(\sqrt{\frac{\kappa }{\kappa +1}}{\tilde{H}}^{IU,L}+\frac{1}{\epsilon }\sqrt{\frac{1}{\kappa +1}}{\tilde{H}}^{IU,NL}\right)$$

with

(15) $$D^{IU,L}=\sqrt{{∥I-u∥}^{2}}.$$

The channel matrix $H^{IM}\left(f\right)$ between the IRS and ME can be expressed as

(16) $$H^{IM}\left(f\right)=\sqrt{\frac{C_{0}{\left(D^{IM,L}\right)}^{-2}}{\sigma }}\cdot \left(\sqrt{\frac{\kappa }{\kappa +1}}{\tilde{H}}^{IM,L}+\frac{1}{\epsilon }\sqrt{\frac{1}{\kappa +1}}{\tilde{H}}^{IM,NL}\right)$$

with

(17) $$D^{IM,L}=\sqrt{{∥I-m∥}^{2}}.$$

To maximize the effective achievable rate at the user station for IRS-assisted coal mine wireless communications, it is assumed that the channel state information (CSI) of the links between the BS and IRS, IRS and User, and IRS and ME is fully known. The BS transmits an effective message *s* with a mean of 0 and unit variance to the User employing beamforming. The transmit beamforming vector for *s* is denoted by $w\in C^{K\times 1}$ with the constraint tr(w)≤P, where *P* represents the total transmit power of the BS. The IRS reflection is modeled as $q≜{\left[q_{1},...,q_{N}\right]}^{T}$, where $q_{n}=\psi_{n}e^{j\theta_{n}}$, $\psi_{n}\in \left[0,1\right]$, and $\theta_{n}\in \left[0,2\pi \right)$ denote the amplitude reflection coefficient and phase shift, respectively, n=1,...,N. For simplicity, we assume that $\psi_{n}=1$ and thus $|q_{n}|=1$, n=1,...,N. Due to severe path loss, the signals reflected by the IRS two or more times can be neglected. Based on the established system model, the receiving signals at the User and ME are given by

(18) $$y_{U}\left(f\right)={\left(H^{IU}\left(f\right)\right)}^{T}Q{\left(H^{BI}\left(f\right)\right)}^{T}ws+n_{U},$$

(19) $$y_{M}\left(f\right)={\left(H^{IM}\left(f\right)\right)}^{T}Q{\left(H^{BI}\left(f\right)\right)}^{T}ws+n_{M},$$

where $Q≜diag\left(q\right)\in C^{N\times N}$ represents a diagonal phase shift matrix, $n_{U}$ and $n_{M}$ denote Gaussian noises at the User and ME with means of zero and variances of $\sigma_{U}^{2}$ and $\sigma_{M}^{2}$, respectively. Therefore, the achievable rates at the User and ME are given by

(20) $$R_{U}\left(f\right)=\log_{2}\left(1+\frac{|{\left(H^{IU}\left(f\right)\right)}^{T}Q{\left(H^{BI}\left(f\right)\right)}^{T}{w|}^{2}}{\sigma_{U}^{2}}\right),$$

(21) $$R_{M}\left(f\right)=\log_{2}\left(1+\frac{|{\left(H^{IM}\left(f\right)\right)}^{T}Q{\left(H^{BI}\left(f\right)\right)}^{T}{w|}^{2}}{\sigma_{M}^{2}}\right).$$

Due to the interference from the ME on the IRS reflection link, the effective achievable rate at the user station is given by

(22) $$R_{eff}\left(f\right)=R_{U}\left(f\right)-R_{M}\left(f\right).$$

Our objective is thus to maximize the effective achievable rate $R_{eff}\left(f\right)$ at the user station by jointly optimizing the transmit beamforming vector w, the IRS phase shift vector q and the horizontal coordinate $I_{x}$ of the IRS. The optimization problem can be formulated as follows.

(23) $$\begin{array}{cc} \; & \underset{w,q,I_{x}}{maximize}\;R_{eff}\left(f\right)\\ s.t.\;\; & C1:tr(w)\leq P,\\ \; & C2:|q_{n}|=1,\forall n,\\ \; & C3:\theta_{n}\in \left[0,2\pi \right),\\ \; & C4:I_{x}\in \left[0,u_{x}\right],\\ \; & C5:f\in [f_{c}-B/2,f_{c}+B/2]. \end{array}$$

Since the objective function $R_{eff}\left(f\right)$ is non-convex with respect to w, q and $I_{x}$, it is not feasible to solve this optimization problem directly with these three variables. To address this problem, we perform alternative joint optimization for $R_{eff}\left(f\right)$ by fixing any two variables of w, q or $I_{x}$ and optimizing the remaining one variable. Jointly optimizing w and q is similar to [13]. Here, we focused on optimizing the horizontal coordinate $I_{x}$ of IRS based on the channel model of a coal mine as described in Section 2.1 and embedded it into the three-step joint optimization to maximize $R_{eff}\left(f\right)$.

## 3. A Three-Step Joint Optimization Algorithm

For simplicity, it is assumed that the working frequency *f* is fixed and the noise powers at the User and ME are the same, i.e., $\sigma_{U}^{2}=\sigma_{M}^{2}=\sigma^{2}$. Compared to the size of the tunnels in the coal mine, the effects of the heights of the BS, ME and User on the signals can be ignored. Based on the system model in Section 2, the horizontal coordinate $I_{x}$ of the IRS can range from 0 to $u_{x}$, which represents the horizontal range of deploying IRSs on the tops of tunnels. According to the optimization problem in Equation (23), we first optimize $I_{x}$ given w, q and then perform three-step joint optimization to maximize $R_{eff}\left(f\right)$.

### 3.1. Optimizing $I_{x}$ Given w and q

Given w and q, the optimization problem in Equation (23) can be transformed into

(24) $$\begin{array}{cc} \; & \underset{I_{x}}{maximize}\;R_{eff}\\ s.t.\;\; & C4:I_{x}\in \left[0,u_{x}\right]. \end{array}$$

By substituting Equations (12)–(16) into the effective achievable rate at the user station in Equations (20)–(22), we can obtain

(25) $$\begin{array}{cc} R_{eff} & =R_{U}-R_{M}\\ \; & =\log_{2}\left(\left(\frac{C_{0}{\left(D^{IU,L}\right)}^{-2}C_{0}{\left(D^{BI,L}\right)}^{-2}}{\sigma^{2}}\right){\left|\left({\left({\tilde{H}}^{IU}\right)}^{T}Q{\left({\tilde{H}}^{BI}\right)}^{T}\right)w\right|}^{2}+\sigma^{2}\right)\\ \; & \;-\log_{2}\left(\left(\frac{C_{0}{\left(D^{IM,L}\right)}^{-2}C_{0}{\left(D^{BI,L}\right)}^{-2}}{\sigma^{2}}\right){\left|\left({\left({\tilde{H}}^{IM}\right)}^{T}Q{\left({\tilde{H}}^{BI}\right)}^{T}\right)w\right|}^{2}+\sigma^{2}\right), \end{array}$$

where ${\tilde{H}}^{BI}=\sqrt{\frac{\kappa }{\kappa +1}}{\tilde{H}}^{BI,L}+\frac{1}{\epsilon }\sqrt{\frac{1}{\kappa +1}}{\tilde{H}}^{BI,NL},\;\;{\tilde{H}}^{IU}=\sqrt{\frac{\kappa }{\kappa +1}}{\tilde{H}}^{IU,L}+\frac{1}{\epsilon }\sqrt{\frac{1}{\kappa +1}}{\tilde{H}}^{IU,NL},\;\;{\tilde{H}}^{IM}=\sqrt{\frac{\kappa }{\kappa +1}}{\tilde{H}}^{IM,L}+\frac{1}{\epsilon }\sqrt{\frac{1}{\kappa +1}}{\tilde{H}}^{IM,NL}.$

  Since $\sigma^{2}$ is much smaller than the left term of "+" in the above equation, it can be ignored. Based on Equations (13)–(17), Equation (25) can be further transformed into

(26) $$\begin{array}{cc} R_{eff}= & R_{U}-R_{M}\\ = & \underset{\gamma_{1}}{\underset{︸}{\log_{2}\left(\frac{\left(C_{0}^{2}/\sigma^{2}\right){\left|({\left({\tilde{H}}^{IU}\right)}^{T}Q{\left({\tilde{H}}^{BI}\right)}^{T})w\right|}^{2}}{\left(I_{z}^{2}+{\left(u_{x}-I_{x}\right)}^{2}\right)\left(I_{z}^{2}+I_{x}^{2}\right)}\right)}}\\ \; & -\underset{\gamma_{2}}{\underset{︸}{\log_{2}\left(\frac{\left(C_{0}^{2}/\sigma^{2}\right){\left|({\left({\tilde{H}}^{IM}\right)}^{T}Q{\left({\tilde{H}}^{BI}\right)}^{T})w\right|}^{2}}{\left(I_{z}^{2}+{\left(m_{x}-I_{x}\right)}^{2}\right)\left(I_{z}^{2}+I_{x}^{2}\right)}\right)}}. \end{array}$$

According to the fact that $\gamma_{1}$ is convex with respect to ${\left(u_{x}-I_{x}\right)}^{2}$ and $I_{x}^{2}$ in Equation (26), the bound of $\gamma_{1}$ can be derived by its first-order Taylor expansion at ${\left(u_{x}-I_{x}^{t}\right)}^{2}$ and ${\left(I_{x}^{t}\right)}^{2}$. Therefore, $\gamma_{1}$ can be expressed as

(27) $$\begin{array}{cc} \gamma_{1} & =\log_{2}\left(\frac{\left(C_{0}^{2}/\sigma^{2}\right){\left|({\left({\tilde{H}}^{IU}\right)}^{T}Q{\left({\tilde{H}}^{BI}\right)}^{T})w\right|}^{2}}{\left(I_{z}^{2}+{\left(u_{x}-I_{x}\right)}^{2}\right)\left(I_{z}^{2}+I_{x}^{2}\right)}\right)\\ \; & =\log_{2}\left(\frac{C_{0}^{2}}{\sigma^{2}}{\left|({\left({\tilde{H}}^{IU}\right)}^{T}Q{\left({\tilde{H}}^{BI}\right)}^{T})w\right|}^{2}\right)+\log_{2}\left(\frac{1}{I_{z}^{2}+{\left(u_{x}-I_{x}\right)}^{2}}\right)+\log_{2}\left(\frac{1}{I_{z}^{2}+I_{x}^{2}}\right)\\ \; & \geq \log_{2}\left(\frac{C_{0}^{2}}{\sigma^{2}}{\left|({\left({\tilde{H}}^{IU}\right)}^{T}Q{\left({\tilde{H}}^{BI}\right)}^{T})w\right|}^{2}\right)\\ \; & \;+\log_{2}\left(\frac{1}{I_{z}^{2}+{\left(u_{x}-I_{x}^{t}\right)}^{2}}\right)+\frac{I_{z}^{2}+{\left(u_{x}-I_{x}^{t}\right)}^{2}}{\ln2}\cdot \left(\frac{-({\left(u_{x}-I_{x}\right)}^{2}-{\left(u_{x}-I_{x}^{t}\right)}^{2})}{{\left(I_{z}^{2}+{\left(u_{x}-I_{x}^{t}\right)}^{2}\right)}^{2}}\right)\\ \; & \;+\log_{2}\left(\frac{1}{I_{z}^{2}+{\left(I_{x}^{t}\right)}^{2}}\right)+\frac{I_{z}^{2}+{\left(I_{x}^{t}\right)}^{2}}{\ln2}\cdot \left(\frac{-(I_{x}^{2}-{\left(I_{x}^{t}\right)}^{2})}{{\left(I_{z}^{2}+{\left(I_{x}^{t}\right)}^{2}\right)}^{2}}\right)\\ \; & =\gamma_{1}^{lowerbd}. \end{array}$$

Next, we derive the bound of $\gamma_{2}$ by introducing two auxiliary variables $\mu_{1}$ and $\mu_{2}$ constrained by

(28) $$\begin{array}{c} \mu_{1}\leq {\left(m_{x}-I_{x}\right)}^{2}, \end{array}$$

(29) $$\begin{array}{c} \mu_{2}\leq I_{x}^{2}. \end{array}$$

Therefore, $\gamma_{2}$ can be expressed as

(30) $$\begin{array}{cc} \gamma_{2} & =\log_{2}\left(\frac{\left(C_{0}^{2}/\sigma^{2}\right){\left|({\left({\tilde{H}}^{IM}\right)}^{T}Q{\left({\tilde{H}}^{BI}\right)}^{T})w\right|}^{2}}{\left(I_{z}^{2}+{\left(m_{x}-I_{x}\right)}^{2}\right)\left(I_{z}^{2}+I_{x}^{2}\right)}\right)\\ \; & =\log_{2}\left(\frac{C_{0}^{2}}{\sigma^{2}}{\left|({\left({\tilde{H}}^{IM}\right)}^{T}Q{\left({\tilde{H}}^{BI}\right)}^{T})w\right|}^{2}\right)+\log_{2}\left(\frac{1}{I_{z}^{2}+{\left(m_{x}-I_{x}\right)}^{2}}\right)+\log_{2}\left(\frac{1}{I_{z}^{2}+I_{x}^{2}}\right)\\ \; & \leq \log_{2}\left(\frac{C_{0}^{2}}{\sigma^{2}}{\left|({\left({\tilde{H}}^{IM}\right)}^{T}Q{\left({\tilde{H}}^{BI}\right)}^{T})w\right|}^{2}\right)+\log_{2}\left(\frac{1}{I_{z}^{2}+\mu_{1}}\right)+\log_{2}\left(\frac{1}{I_{z}^{2}+\mu_{2}}\right)\\ \; & =\gamma_{2}^{upperbd}. \end{array}$$

Since the constraints on $\mu_{1}$ and $\mu_{2}$ in Equations (28) and (29) are non-convex, we use their first-order Taylor expansions to transform them into convex constraints.

(31) $$\mu_{1}\leq {\left(m_{x}-I_{x}^{t}\right)}^{2}+2\cdot \left(m_{x}-I_{x}^{t}\right)\cdot \left(I_{x}-I_{x}^{t}\right),$$

(32) $$\mu_{2}\leq {\left(I_{x}^{t}\right)}^{2}+2\cdot I_{x}^{t}\cdot \left(I_{x}-I_{x}^{t}\right).$$

Based on these above transformations, the optimization problem for the horizontal deployment position of the IRS in Equation (24) can be transformed into the following convex optimization problem.

(33) $$\begin{array}{cc} \underset{I_{x},\mu_{1},\mu_{2}}{maximize}\; & \gamma =\gamma_{1}^{lowerbd}-\gamma_{2}^{upperbd}\\ s.t.\;\; & C1:I_{x}\in \left[0,u_{x}\right],\\ \; & C2:\mu_{1},\mu_{2}. \end{array}$$

The complete solution with an iterative loop is organized in Algorithm 1, where $I_{x}^{t}$ denotes the horizontal coordinate of the IRS in the *t*-th iteration.
**Algorithm 1** Optimization Scheme for the Horizontal Position of IRS1:Initialize the optimized transmit beamforming vector $w^{opt}$ of BS and the phase shift $q^{opt}$ of IRS.2:Initialize the horizontal coordinate of IRS as $I_{x}^{0}$ and set t=0. Denote the convergence threshold as $\xi_{1}$.3:**repeat**4:   The solution of the horizontal coordinate $I_{x}$ is obtained by solving   $\begin{array}{l} \underset{I_{x},\mu_{1},\mu_{2}}{max}\gamma =\gamma_{1}^{lowerbd}-\gamma_{2}^{upperbd}\\ \;{s.t.\;\;\;C1:∥w∥}^{2}\leq P,\\ \;C2:|q_{n}|=1,\;\forall n,\\ \;C3:I_{x}\in \left[0,u_{x}\right],\\ \;C4:\mu_{1},\mu_{2}. \end{array}$5:   t=t+1.6:   $I_{x}^{t}=I_{x}.$7:**until** $|R_{eff}^{t}-R_{eff}^{t-1}|\leq \xi_{1}.$

### 3.2. Three-Step Joint Optimization with w, q and $I_{x}$

The solutions to the optimization problem in Equation (23) can be divided into three steps as shown in Algorithm 2, which optimize the transmit beamforming vector w, the phase shift q and the horizontal coordinate $I_{x}$ of the IRS jointly and alternately. The transmit beamforming vector w of the BS can be optimized using the normalized eigenvector corresponding to the largest eigenvalue and the optimal phase shift q can be obtained by using the BCAM algorithm proposed in [13]. The horizontal deployment position $I_{x}$ of the IRS can be optimized by applying the proposed Algorithm 1. The alternate joint optimization is performed by fixing two variables of w, q or $I_{x}$ and optimizing the remaining one.
**Algorithm 2** Alternate Joint Optimization Scheme1:Initialize the transmit beamforming vector w of BS, the phase shift q of IRS, the horizontal coordinate $I_{x}^{0}$ of IRS and set t=0. Denote the convergence threshold as $\xi_{2}$.2:**repeat**3:   Fix q and $I_{x}^{t}$, and optimize $w\to w^{opt}$,4:   Fix $w^{opt}$ and $I_{x}^{t}$, and optimize $q\to q^{opt}$,5:   Fix $w^{opt}$ and $q^{opt}$, and optimize $I_{x}\to I_{x}^{opt}$.6:   t=t+1.7:   $q=q^{opt}.$8:   $I_{x}^{t}=I_{x}^{opt}.$9:**until** $|R_{eff}^{t}-R_{eff}^{t-1}|\leq \xi_{2}.$

## 4. Simulation Results

In this section, we perform different simulations to demonstrate the effectiveness of the proposed three-step optimization scheme based on the channel model of a coal mine. The parameter configurations are listed in Table 1 and here we set the working frequency to be 915 MHz, a commonly used commercial working frequency in coal mine wireless communication systems, as pointed out in [17,20].

**Figure 3:**
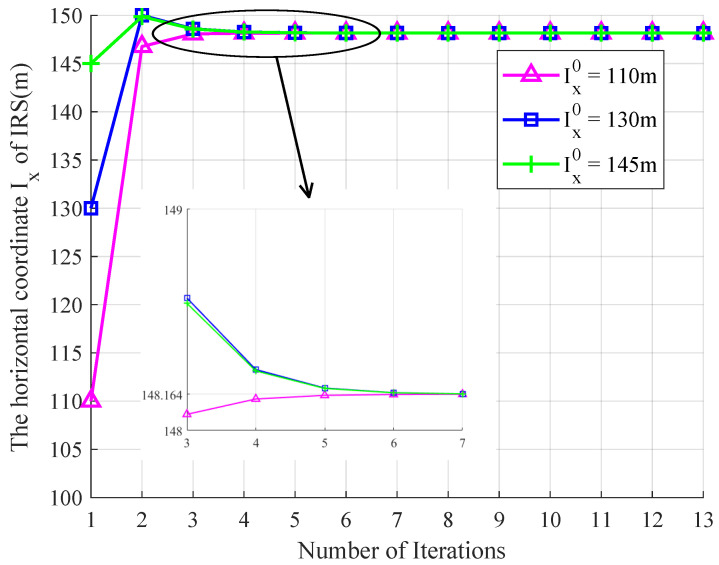
Convergence analysis under different initial horizontal coordinates of IRS with P=15 dBm and $\delta_{h}=0.02$.

We first simulated the effects of different initial horizontal deployment positions on the convergence to the optimal horizontal deployment position using the horizontal coordinate optimization algorithm proposed in Section 3.1 with the total transmit power P=15 dBm and the roughness $\delta_{h}=0.02$ as shown in Figure 3. The three initial horizontal coordinate $I_{x}^{0}$ were set to be 110 m, 130 m, and 145 m, respectively. From Figure 3, it can be seen that when the number of iterations was increased to about 7, these three curves all convergd to the same optimal horizontal coordinate $I_{x}^{opt}=148.16$ m, which indicates that the optimized horizontal coordinate obtained by the proposed optimization algorithm is independent of the initial deployment positions of the IRS.

**Figure 4:**
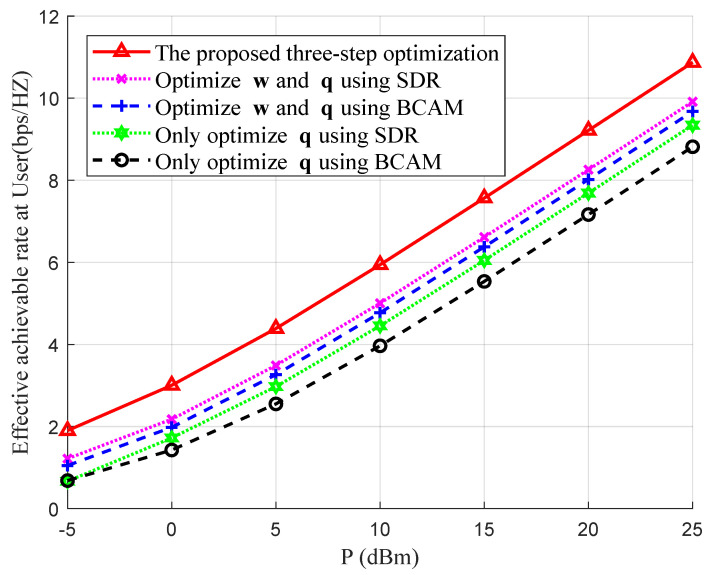
Comparisons of the effective achievable rates at the user station between different rate optimization schemes with varying *P* and fixed $\delta_{h}=0.02$.

Next, we compared the effective achievable rates at the user using different rate optimization schemes, including the proposed three-step joint rate optimization algorithm, the SDR algorithm in [11] and the BCAM algorithm in [13] under the coal mine channel model, as shown in Figure 4. It can be observed that the effective achievable rates obtained by the proposed three-step joint rate optimization scheme are the highest. Compared to the SDR algorithm and the BCAM algorithm, which optimized w and q alternately, the effective achievable rates could be improved by approximately 14.39% and 18.64% when varying the total transmit power *P* from −5 dBm to 25 dBm, respectively. In addition, 25.09% and 36.69% improvements were obtained in comparison with these two algorithms which only optimized q without beamforming at BS. The simulation results verify the necessities of optimizing the deployment position of the IRS in coal mines and the effectiveness of the proposed three-step joint rate optimization scheme.

**Figure 5:**
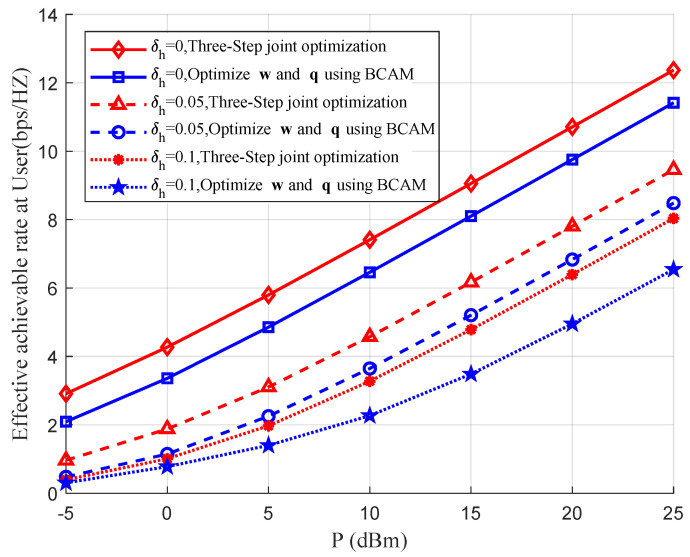
Effects of the roughness of the tunnel walls in the coal mine on the effective achievable rates at the user station using different rate optimization schemes.

In the following section, we investigated the effects of the roughness of the tunnels in a coal mine on the effective achievable rates for users benefiting from the proposed three-step joint rate optimization scheme and the proposed alternate optimization of w and q in [13], as shown in Figure 5. Here, we chose three typical values of roughness, 0, 0.05 and 0.1, which means that the tunnels were completely smooth, moderately rough and very rough, respectively. The simulation results show that as the roughness $\delta_{h}$ increases, the effective achievable rates at the user station, as expected, decrease for both rate optimization schemes. However, regardless of the roughness, the effective achievable rates obtained by using the proposed three-step rate optimization scheme are always higher than those obtained by the scheme in [13], which indicates that the proposed three-step joint rate optimization scheme which includes the optimization of the deployment position of the IRS performs better in the coal mine scenario.

**Figure 6:**
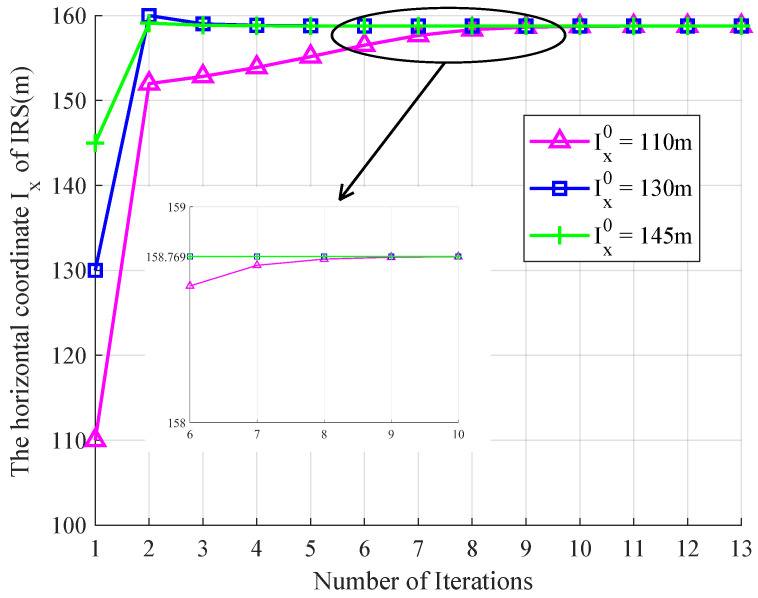
Comparisons of the effective achievable rates at the user station between different rate optimization schemes with varied *P* and fixed $\delta_{h}=0.02$.

**Figure 7:**
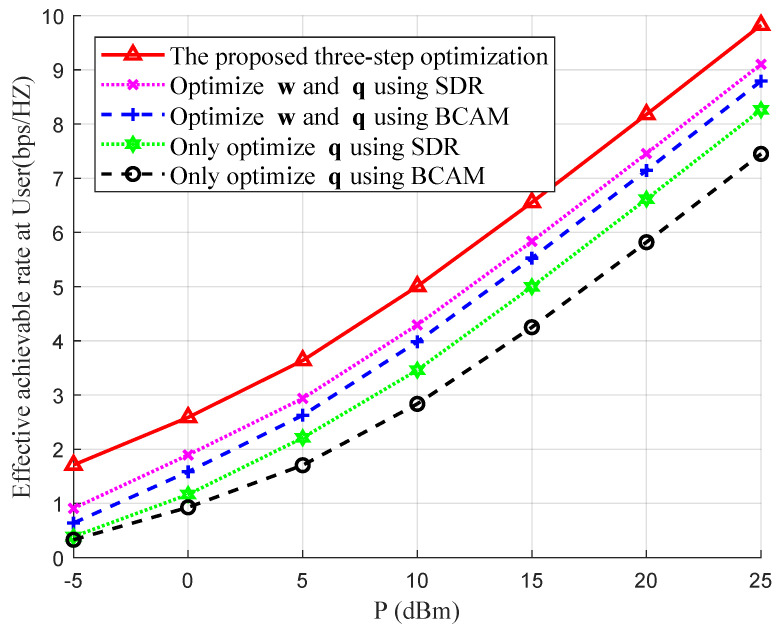
Comparisons of the effective achievable rates at the user station between different rate optimization schemes with varied *P* and fixed $\delta_{h}=0.02$.

Finally, in order to further verify the effectiveness and generality of the proposed three-step joint optimization algorithm, we conducted supplementary simulations for another working frequency in coal mine wireless communication systems. We set the working frequency and carrier frequency to be f=2.45 GHz and $f_{c}=3$ GHz, and increased the distance between the User and BS to 160 m. The convergence to the optimal IRS deployment horizontal position is shown in Figure 6. We can obtain the similar conclusion that, by increasing the number of iterations to about 9, these three curves all converge to the same optimal horizontal coordinate $I_{x}^{opt}=158.77$ m, regardless of the initial deployment positions of the IRS. Moreover, the effective achievable rates at the user station using these three different rate optimization schemes under the coal mine channel model are also plotted in Figure 7. The simulation results show that when varying the total transmit power *P* from −5 dBm to 25 dBm, 12.32% and 18.62% improvements could be obtained compared with those that alternately optimized w and q, while 31.16% and 54.17% improvements could be obtained compared with those that only optimized q.

## 5. Conclusions

This paper established an IRS-assisted coal mine communication system modeled by a 3D MIMO GBSM, for which a three-step joint optimization involving the transmit power, the IRS phase shifts and the IRS deployment position was proposed to maximize the effective achievable rate at the user station. To solve the non-convexity of the optimization problem for the IRS deployment position, logarithmic operations, Taylor relaxation and two auxiliary variables were introduced to transform it into a convex problem. The simulation results show that the proposed three-step joint rate optimization scheme performed better in the coal mine scenario regardless of the roughness of the tunnels, and the convergence to the optimal deployment position of the IRS was independent of the initial deployment position. By comparison, approximately 12.32% to 54.17% improvements in the effective achievable rates at the user station could be obtained by using the proposed rate optimization scheme under different parameter configurations. Our work provided the feasibility of deploying an IRS to enhance the performance of coal mine wireless communication systems and verified the necessity to optimize the IRS deployment position to improve the effective achievable rates at the user station.

## Figures and Tables

**Table 1:** Parameterconfigurations for simulations.

System Parameters	Value
Number of BS antennas, K	4
Number of IRS reflection elements, $N_{x}\times N_{y}$	8×8
ME coordinate, $\left[m_{x},m_{y},m_{z}\right]$	[145 m, 0, 0]
User coordinate, $\left[u_{x},u_{y},u_{z}\right]$	[150 m, 0, 0]
IRS vertical coordinate, $I_{z}$	5 m
Normalized path loss, $C_{0}$	−30 dB
Noise standard deviation, σ	−80 dBm
Carrier frequency, $f_{c}$	1.2 GHz
Working frequency, *f*	915 MHz

## Data Availability

Data are contained within the article.

## References

[1] Ranjany A., Misraz P., Dwivediz B., Sahuy H.B. Channel modeling of wireless communication in underground coal mines. Proceedings of the 8th International Conference on Communication Systems and Networks (COMSNETS).

[2] Ben Mabrouk I., Talbi L., Nedil M., Hettak K. (2012). MIMO-UWB Channel Characterization Within an Underground Mine Gallery. IEEE Trans. Antennas Propag..

[3] de Oliveira Gomes P.H., Guieiro G., de Almeida E.P.L., Garcia L.G.U. Evaluation of Shadowing Caused by Mining Machinery in V2I Communications. Proceedings of the IEEE 29th Annual International Symposium on Personal, Indoor and Mobile Radio Communications (PIMRC).

[4] Játiva P.P., Azurdia-Meza C.A., Sánchez I., Seguel F., Zabala-Blanco D., Firoozabadi A.D., Gutiérrez C.A., Soto I. (2020). A VLC Channel Model for Underground Mining Environments With Scattering and Shadowing. IEEE Access.

[5] ElMossallamy M.A., Zhang H., Song L., Seddik K.G., Han Z., Li G.Y. (2020). Reconfigurable Intelligent Surfaces for Wireless Communications: Principles, Challenges, and Opportunities. IEEE Trans. Cogn. Commun. Netw..

[6] Liu Y., Liu X., Mu X., Hou T., Xu J., Di Renzo M., Al-Dhahir N. (2021). Reconfigurable Intelligent Surfaces: Principles and Opportunities. IEEE Commun. Surveys Tut..

[7] Zhang Z., Dai L., Chen X., Liu C., Yang F., Schober R., Poor H.V. (2023). Active RIS vs. Passive RIS: Which Will Prevail in 6G?. IEEE Trans. Commun..

[8] Wang B., Yuan Z., Lu J., Zhang X. (2024). Multitask Collaborative Learning Neural Network for Radio Signal Classification. IEEE Trans. Commun..

[9] Yuan X., Zhang Y.-J.A., Shi Y., Yan W., Liu H. (2021). Reconfigurable-Intelligent-Surface Empowered Wireless Communications: Challenges and Opportunities. IEEE Wirel. Commun..

[10] Kisseleff S., Chatzinotas S., Ottersten B. (2021). Reconfigurable Intelligent Surfaces in Challenging Environments: Underwater, Underground, Industrial and Disaster. IEEE Access.

[11] Cui M., Zhang G., Zhang R. (2019). Secure Wireless Communication via Intelligent Reflecting Surface. IEEE Wireless Commun. Lett..

[12] Song Y., Khandaker M.R.A., Tariq F., Wong K.-K., Toding A. Truly Intelligent Reflecting Surface-Aided Secure Communication Using Deep Learning. Proceedings of the IEEE 93rd Vehicular Technology Conference (VTC2021-Spring).

[13] Kumar V., Flanagan M.F., Kwan Ng D.W., Tran L.-N. On the Secrecy Rate under Statistical QoS Provisioning for RIS-assisted MISO Wiretap Channel. Proceedings of the IEEE Global Communications Conference (GLOBECOM).

[14] Wu Q., Zhang S., Zheng B., You C., Zhang R. (2021). Intelligent Reflecting Surface-Aided Wireless Communications: A Tutorial. IEEE Trans. Commun..

[15] Ren Y., Zhou R., Teng X., Meng S., Zhou M., Tang W., Li X., Li C., Jin S. (2023). On Deployment Position of RIS in Wireless Communication Systems: Analysis and Experimental Results. IEEE Wirel. Commun. Lett..

[16] Liu Y., Zhang J., Zhang Y., Gong H., Jiang T., Liu G. (2024). How to Extend 3-D GBSM to Integrated Sensing and Communication Channel With Sharing Feature?. IEEE Wirel. Commun. Lett..

[17] Ji X., Wang C.-X., Chang H. A Novel MIMO Channel Model for Underground Mine Communications. Proceedings of the IEEE/CIC International Conference on Communications in China (ICCC).

[18] Gong H., Zhang J., Zhang Y., Zhou Z., Liu G. (2024). How to Extend 3-D GBSM to RIS Cascade Channel With Non-Ideal Phase Modulation?. IEEE Wirel. Commun. Lett..

[19] Zhou C. (2017). Ray Tracing and Modal Methods for Modeling Radio Propagation in Tunnels With Rough Walls. IEEE Trans. Antennas Propag..

[20] Gangwar K., Chen G.C.Y., Chan K.K.M., Gangwar R.K., Rambabu K. (2021). Antenna System for Communication in Underground Mining Environment to Ensure Miners Safety. IEEE Access.

